# Clinical–Radiological Spectrum of Cerebral Amyloid Angiopathy‐Related Inflammation

**DOI:** 10.1002/ana.78029

**Published:** 2025-09-19

**Authors:** Larysa Panteleienko, Gargi Banerjee, Dermot Mallon, Kitti Thiankhaw, Rupert Oliver, Victoria Harvey, Gareth Ambler, Michael Zandi, Hans Rolf Jäger, David J. Werring

**Affiliations:** ^1^ Stroke Research Center, Department of Translational Neuroscience and Stroke UCL Queen Square Institute of Neurology London UK; ^2^ Deptartment of Neurology Bogomolets National Medical University Kyiv Ukraine; ^3^ MRC Prion Unit at UCL, Institute of Prion Diseases London UK; ^4^ Comprehensive Stroke Service National Hospital for Neurology and Neurosurgery, Queen Square, University College London Hospitals NHS Foundation Trust London UK; ^5^ Lysholm Department of Neuroradiology National Hospital for Neurology and Neurosurgery London UK; ^6^ Division of Neurology, Department of Internal Medicine, Faculty of Medicine Chiang Mai University Chiang Mai Thailand; ^7^ Department of Statistical Science UCL Queen Square Institute of Neurology London UK; ^8^ Department of Neuroinflammation UCL Queen Square Institute of Neurology London UK; ^9^ Neuroradiological Academic Unit, Department of Translational Neuroscience and Stroke UCL Queen Square Institute of Neurology London UK

## Abstract

**Objective:**

To identify clinical and radiological features of cerebral amyloid angiopathy‐related inflammation (CAA‐ri), and compare these features with those of sporadic CAA, to improve the understanding, diagnosis, and clinical care of CAA‐ri.

**Methods:**

We retrospectively reviewed routine clinical data from 37 patients with CAA‐ri and 158 patients with sporadic CAA, including conventional vascular risk factors and comorbidities. We assessed brain magnetic resonance imaging for: radiological markers of CAA; features of amyloid‐related imaging abnormalities with edema/effusion (ARIA‐E) including parenchymal white matter hyperintensities, sulcal hyperintensities, and gyral swelling; and evidence of neurodegeneration (medial temporal atrophy and global cortical atrophy).

**Results:**

Compared with patients with sporadic CAA, patients with CAA‐ri had more numerous lobar cerebral microbleeds (median 207[IQR 33‐811] vs 19[IQR 7‐58], *p* < 0.001), and higher rates of medial temporal and global cortical atrophy. In comparison with sporadic CAA, all ARIA‐E features were much more common in patients with CAA‐ri (parenchymal hyperintensities 89% vs 3%, sulcal hyperintensities 78% vs 9%, and gyral swelling 86% vs 0.6%), as were conventional vascular risk factors (hypertension, dyslipidemia) and long‐term comorbidities (inflammatory and infectious disorders, autoimmune or connective tissue disorders, or malignancies).

**Interpretation:**

Features of ARIA‐E (parenchymal white matter hyperintensities, sulcal hyperintensities, and gyral swelling) are more common in CAA‐ri in comparison with “non‐inflammatory” sporadic CAA, suggesting shared mechanisms with Alzheimer's disease immunotherapy and a potential role in improving diagnostic accuracy for CAA‐ri. The high prevalence of atrophy and lobar cerebral microbleeds suggests a potential mechanistic role for capillary CAA, Alzheimer's disease, or both, in CAA‐ri. Cardiovascular risk factors and other long‐term comorbidities may also be relevant to the underlying mechanisms of CAA‐ri. ANN NEUROL 2026;99:148–158

Cerebral amyloid angiopathy‐related inflammation (CAA‐ri) describes a clinical and radiological syndrome attributed to spontaneous inflammatory reaction to amyloid‐β deposition in the walls of small‐to‐medium‐sized leptomeningeal and cortical vessels (cerebral amyloid angiopathy [CAA]).^1^ However, the mechanisms underlying the inflammatory response remain unknown, and attempts to uncover them are hampered by the relative rarity of CAA‐ri.^2^, ^3^ Although several reports, case series, and systematic reviews of CAA‐ri cases exist, large, well‐phenotyped cohorts remain rare.^4^

Although classically CAA‐ri is characterized clinically by subacute cognitive decline, altered mental state, headaches, and seizures,^2^, ^5^ other recognized presenting symptoms include transient focal neurological deficits or stroke‐like events without encephalopathy,^6^, ^7^ in addition to patients with radiological evidence of CAA‐ri in the absence of significant clinical symptoms.^8^, ^9^, ^10^, ^11^ Case reports are often susceptible to bias, with more unusual cases more likely to be reported, so there remains an unmet need to review clinical presentations of CAA‐ri in less selective cohorts.

The radiological diagnosis of CAA‐ri usually requires magnetic resonance imaging (MRI), with emphasis on the presence of dynamic confluent brain parenchymal white matter changes. These changes are often asymmetric, but can be unifocal or multifocal, cortico‐subcortical or deep, and are sometimes associated with mass effect.^12^, ^13^ More recently, other distinct radiological features have been described in patients CAA‐ri, including sulcal hyperintensities, gyral swelling, leptomeningeal enhancement, and multiple diffusion‐weighted imaging hyperintense lesions.^14^, ^15^, ^16^ Sulcal hyperintensities and gyral swelling are of particular interest, as they are also features of amyloid‐related imaging abnormalities with edema/effusion (ARIA‐E), which occurs in some patients after treatment with anti‐amyloid‐β (Aβ) immunotherapy for Alzheimer's disease, and which, like CAA‐ri, is hypothesized to be an immune‐mediated attempt to clear parenchymal Aβ.^17^, ^18^ Investigation of these potential new CAA‐ri markers in larger cohorts could facilitate understanding and improve the diagnosis of CAA‐ri in those with atypical clinical presentations, potentially reducing the requirement for confirmatory brain biopsy.^19^

The objective of this project was to systematically review data from a cohort of consecutive patients diagnosed with CAA‐ri, and compare them with patients with “non‐inflammatory” sporadic CAA to identify clinical and radiological features to support early diagnosis and better understand potential mechanisms of inflammation. We additionally reviewed follow‐up data to guide decisions about long‐term monitoring and treatment of CAA‐ri.

## Materials and Methods

### Patient Selection

This is a retrospective observational review of clinical and radiological data from patients evaluated by the Comprehensive Stroke Service at the National Hospital for Neurology and Neurosurgery, Queen Square, London. This specialist service receives referrals from North Central London and other neurology services throughout the UK.

For CAA‐ri, we included all consecutive patients who met “definite” (ie, biopsy‐proven)^13^ or “probable” diagnostic criteria,^12^ either at first presentation or during subsequent follow‐up, between March 2013 and December 2023. All alternative diagnoses, such as cerebral vasculitis, neoplasms, infectious, and metabolic diseases, were excluded after thorough evaluation. All patients with suspected or confirmed CAA‐ri undergo a standardized and comprehensive evaluation as part of clinical care, supported by the Queen Square specialist intracranial hemorrhage clinic. For comparison, we included all patients consecutively referred to our intracranial haemorrhage clinic between February 2016 and December 2024 who were diagnosed with “non‐inflammatory” sporadic CAA, all of whom met probable Boston 2.0 clinical–radiological criteria for CAA, but without any typical radiological features suggesting CAA‐ri.^12^

### Approvals

The project's overall objective is to improve the diagnosis and clinical care of patients with CAA‐ri. The protocol was approved as a service evaluation by the National Hospital for Neurology and Neurosurgery Quality and Safety Team (07‐202324‐SE), providing permission to use data collected during the routine care of patients with CAA to improve clinical service provision. Therefore, the study was judged not to require approval from an Institutional Review Board, and the requirement for written informed consent was waived.

### Clinical Data

We reviewed all available data collected during the provision of routine clinical care and documented in medical records. Previous functional status was determined by using information from medical records according to the Specifications Manual for Joint Commission National Quality Measures (v2023B)^21^ to retrospectively define the modified Rankin Scale (mRS) before CAA‐ri diagnosis.

We recorded vascular and non‐vascular comorbidities and vascular risk factors; we classified long‐term non‐vascular comorbidities as infectious and inflammatory conditions, autoimmune and connective tissue disorders, and malignancies.

In our service, all patients with CAA‐ri are reviewed at least annually (and more frequently if there are new or evolving symptoms). Follow‐up reviews are usually in‐person appointments and include standardized functional status assessment including the mRS, details of new clinical symptoms, repeat imaging, and mortality, according to clinical indications. Clinical–radiological relapse was defined as the occurrence of neurological symptoms and worsening MRI imaging features consistent with CAA‐ri at least 3 months after the resolution of their first (or most recent) episode of inflammation; radiological relapse was defined similarly when there was radiological evidence of new CAA‐ri disease activity without clinical symptoms.

### Imaging

MRI images in the Comprehensive Stroke Service at the National Hospital for Neurology and Neurosurgery were acquired on 3T scanners with a standardized MRI protocol, which includes T1‐weighted, T2‐weighted, fluid‐attenuated inversion recovery (FLAIR), diffusion‐weighted imaging, and blood‐sensitive (historically T2*‐weighted gradient recalled echo; now susceptibility weighted imaging, SWI) sequences for all patients. Three of 37 patients with CAA‐ri and 1 of 152 patients with sporadic “non‐inflammatory” CAA were assessed with T2*‐weighted gradient recalled echo images. Additional sequences, such as contrast‐enhanced T1‐weighted images, were acquired at the treating clinician's discretion.

All imaging was evaluated by a neurovascular clinician (L.P.), with support and review by experienced neuroradiologists (D.M. or H.R.J.) where needed.

Hemorrhagic and non‐hemorrhagic radiological markers, as defined by the Boston 2.0 criteria^20^ for “non‐inflammatory” sporadic CAA, were collected for all patients. Hemorrhagic markers included acute or previous intracerebral hemorrhage (ICH), acute convexity subarachnoid hemorrhage, cortical superficial siderosis, and strictly lobar cerebral microbleeds. Non‐hemorrhagic markers included white matter hyperintensities in a “multispot” pattern and severe (>20) MRI‐visible perivascular spaces in the centrum semiovale.

We recorded imaging features associated with amyloid‐related imaging abnormalities (ARIA), a phenomenon observed in some patients with Alzheimer's disease treated with anti‐amyloid immunotherapies, using the Barkhof Grand Total Scale (BGTS) for ARIA with edema/effusions (ARIA‐E).^17^ The BGTS includes 3 features: (1) parenchymal white matter hyperintensities, defined as a hyperintense signal in white matter on T2/FLAIR images sometimes with evidence of mass effect, which represents vasogenic edema; (2) sulcal hyperintensity, defined as the failure of the suppression of cerebrospinal fluid (CSF) signal within a sulcus on FLAIR images and not related to acute subarachnoid hemorrhage; and (3) gyral swelling, defined as the enlargement of a region of cerebral hemispheres causing partial or complete sulcal effacement. To test interrater reliability, 10 MRI scans were reviewed by the neurovascular clinician (L.P.) and experienced neuroradiologist (D.M.), and outcomes were compared using Kappa statistics. There was good interrater agreement: parenchymal white matter hyperintensities, κ = 0.72, with 82.5% agreement; sulcal hyperintensity, κ = 0.83, with 93.8% agreement; and gyral swelling, κ = 0.61, with 82.5% agreement.

We reviewed contrast‐enhanced imaging for evidence of leptomeningeal or parenchymal enhancement and assessed medial temporal atrophy assessed using the Scheltens' visual scale^22^ on coronal T1‐weighted images (score 0 to 4), recording the most affected side, and global cortical atrophy assessed using the Pasquier visual scale^23^ on axial T1 or FLAIR images (score 0 to 3).

### Statistical Analysis

Analyses were performed by L.P. using Stata18 (StataCorp, College Station, TX, USA). We calculated descriptive statistics using numbers and percentages for discrete variables, mean with standard deviation for normally distributed continuous data, and median with interquartile range (IQR) for non‐normal continuous variables. Comparisons were obtained with the χ^2^‐test or analysis of variance for categorical and continuous variables, respectively. A *p* value of <0.05 was considered statistically significant.

## Results

### Baseline Characteristics

We included 37 patients with CAA‐ri (9/37 were biopsy‐proven and 28/37 met radiological criteria for probable CAA‐ri) and 158 patients with “non‐inflammatory” sporadic CAA (Table 1). One patient with CAA‐ri and 4 patients with sporadic CAA were excluded due to missing some of the required MRI sequences. The age and sex distributions were similar in both groups. In comparison with patients with “non‐inflammatory” sporadic CAA, patients with CAA‐ri were more likely to have conventional vascular risk factors, specifically hypercholesterolemia (86% vs 32%, *p* < 0.001), and hypertension (78% vs 55%, *p* = 0.009). Patients with CAA‐ri were also more likely to have non‐vascular long‐term medical conditions (73% vs 41%), with more than one‐third of patients of CAA‐ri having ≥2 such conditions (35%, compared with 11%, *p* < 0.001 in the “non‐inflammatory” sporadic CAA group); these included conditions associated with a persistent infection or inflammatory state (49% vs 22%, *p* < 0.001), autoimmune or connective tissue disorders (38% vs 20%, *p* = 0.018), and malignancies (27% vs 12%, *p* = 0.021; Table 1).

**TABLE 1:** Baseline Clinical Characteristics

	CAA‐ri	“Non‐inflammatory” sporadic CAA	Difference in proportions or means (95% CI)	*p*
No. patients	37	158		
Mean age, years (SD)	70.9 (7.9)	73.0 (7.0)	−29.7 (−65.6 to 6.2)	0.105
Sex, male, *n* (%)	21 (57%)	90 (57%)	0.2 (−17.5 to 17.9)	0.981
Cerebrovascular risk factors, *n* (%)				
Hypercholesterolemia*	32 (86%)	50 (32%)	54.8 (41.7 to 68.0)	<0.001
Hypertension*	29 (78%)	87 (55%)	23.3 (7.9 to 38.7)	0.009
Current and ex‐smokers	11 (30%)	31 (20%)	10.1 (−5.9 to 26.1)	0.178
Alcohol misuse	11 (30%)	33 (21%)	8.8 (−7.2 to 24.9)	0.246
Diabetes	7 (19%)	18 (11%)	7.5 (−6.0 to 21.1)	0.217
Atrial fibrillation	4 (11%)	24 (15%)	4.4 (−15.8 to 7.1)	0.494
Previous ischemic stroke, *n* (%)	5 (14%)	19 (12%)	1.5 (−10.6 to 13.6)	0.804
Ischemic heart disease, *n* (%)	7 (19%)	36 (23%)	9.3 (−22.1 to 3.5)	0.212
Non‐vascular comorbidities, *n* (%)				
Conditions potentially associated with a persistent infection or inflammatory state^a^, *	18 (49%)	35 (22%)	25.2 (8.7 to 41.6)	<0.001
Autoimmune conditions and connective tissue disorders^b^, *	14 (38%)	31 (20%)	18.2 (1.4 to 35.0)	0.018
Malignancies^c^, *	10 (27%)	19 (12%)	15.0 (−0.2 to 30.2)	0.021
At least 1 comorbidity*	27 (73%)	65 (41%)	31.8 (15.6 to 48.1)	<0.001
≥2 comorbidities*	13 (35%)	17 (11%)	24.4 (8.3 to 40.5)	<0.001

* Indicates a statistically significant difference.

^a^ In this cohort, we observed the following relevant diagnoses: long‐standing infectious and inflammatory conditions, chronic obstructive pulmonary disease, recurrent urinary tract infections, advanced stage of osteoarthritis, gout, chronic sinusitis, latent syphilis, and chronic hepatitis C.

^b^ We observed the following autoimmune diseases and connective tissue disorders (in order of frequency): rheumatoid arthritis, autoimmune thyroiditis, giant cell arteritis, scleroderma, Sjögren's syndrome, and systemic lupus erythematosus.

^c^ Observed cancers included (in order of frequency): prostate, breast, bladder, colon, multiple myeloma, and diffuse B‐cell lymphoma.

CAA = cerebral amyloid angiopathy; CAA‐ri = cerebral amyloid angiopathy‐related inflammation; SD = standard deviation.

### Clinical Characteristics

Presenting symptoms of CAA‐ri included encephalopathy in 30 patients (81%, of whom 6 patients had delirium); focal neurological deficit in 18 (49%) patients (of whom 6/18 had transient focal neurological episodes, 5/18 had acute ICH, 1/18 had ischemic stroke, and 6/18 had other focal symptoms related to the area of inflammation – e.g., hemianopia, hemiparesis, neglect); headache in 15 (41%), and seizures in 9 (24%) patients. Most of the patients had more than 1 presenting symptom.

A total of 27 (73%) patients had CSF investigations, which showed non‐specific changes: 4 of 27 had mild cytosis (≤10 cells, lymphocytes), and 18 of 27 had elevated protein levels of 1.20 ± 0.72 g/L. No correlation was found between these data and radiological findings.

A total of 31(84%) received immunosuppressive treatment within 1 week after diagnosis. Thirty patients (81%) had steroid therapy, of whom 27 (73%) had intravenous pulsed therapy with 1 g methylprednisolone for 3–5 days with subsequent oral prednisolone tapered for 6 months (IQR 4.5–7 months); 3 (8%) had “pulse‐therapy” only. One patient (3%) received mycophenolate mofetil due to a relative contraindication to steroid therapy (severe osteoporosis). Three of 30 patients who received steroid therapy also received cyclophosphamide.

Before CAA‐ri diagnosis, 19% of patients (n = 7) were reported to have had a moderate to severe disability, as defined by the mRS (≥3): 2 of 7 due to prior stroke, 2 of 7 due to prior ICH, and 1 patient each with Parkinson's disease, alcoholic polyneuropathy, and severe rheumatoid arthritis.

Follow‐up data were available for 36 of 37 (97%) patients (median follow‐up time 22 months [IQR 12–18.5 months]). Within 1 year, most patients (26/36, 72%) made a complete functional recovery (ie, back to their baseline mRS before CAA‐ri diagnosis), 19% (n = 7) acquired new and persistent disability at 1 year (decline in mRS of ≥2 points). Overall, 11 of 37 patients died, but only 3 (8%) died within 1 year with death attributable to the CAA‐ri diagnosis.

We observed at least 1 CAA‐ri relapse in 12 patients (33%), which occurred within 1 year of CAA‐ri diagnosis in 10 of 36 (28%) patients. Most relapses were both clinical and radiological (n = 8 [22%]; n = 7 within the first year), with asymptomatic radiological relapse observed in a minority (n = 4 [11%]; n = 3 within the first year).

### Radiological Characteristics

The neuroimaging characteristics of patients with CAA‐ri and “non‐inflammatory” sporadic CAA are presented in Table 2.

**TABLE 2:** Baseline Radiological Characteristics

	CAA‐ri	“Non‐inflammatory” sporadic CAA	Difference in proportions and means (95% CI)	*p*
No. patients	37	158		
MRI features of ARIA‐E, *n* (%)				
Parenchymal white matter hyperintensities*	33 (89%)	5 (3%)	86.0 (75.6 to 96.4)	<0.001
Gyral swelling*	32 (86%)	1 (0.6%)	‐	<0.001
Sulcal hyperintensities^a^, *	28 (78%)	15 (9%)	68.3 (54.0 to 82.6)	<0.001
Leptomeningeal contrast enhancement^b^	17 (71%)	n/a	‐	‐
Imaging data for CAA Boston 2.0 criteria and brain atrophy scores, *n* (%)				
Intracerebral hemorrhage*	8(22%)	85 (54%)	−32.2 (−47.6 to −16.8)	<0.001
Convexity subarachnoid hemorrhage*	4 (11%)	44 (28%)	−17.0 (−29.2 to −4.8)	0.030
Lobar cerebral microbleeds, median (IQR)*	207 (33–811)	19 (7–58)	‐	<0.001
Right frontal lobe	8 (1–103)	1 (0–5)	‐	‐
Left frontal lobe	5 (0–33)	1 (0–6)	‐	‐
Right temporal lobe	32 (3–130)	2 (0–7)	‐	‐
Left temporal lobe	25 (2–64)	2 (0–7)	‐	‐
Right parietal lobe	26 (1–111)	2 (0–7)	‐	‐
Left parietal lobe	19 (1–99)	2 (0–8)	‐	‐
Right occipital lobe	32 (4–114)	2 (0–7)	‐	‐
Left occipital lobe	15 (2–101)	2 (0–7)	‐	‐
Cortical superficial siderosis*	14 (38%)	117 (74%)	−36.2 (−53.3 to −19.2)	<0.001
None/focal/disseminated*	23 (62%)/ 8 (22%)/ 6 (16%)	41 (26%)/ 34 (21.5%)/ 83 (52.5%)	‐	<0.001
Multispot white matter hyperintensities	11 (30%)	74 (47%)	−17.1 (−33.8 to −0.4)	0.059
Small diffusion‐weighted imaging lesions*	13 (35%)	27 (17%)	18.0 (1.6 to 34.5)	0.014
Enlarged perivascular spaces in the centrum semiovale (3–4 points)	24 (65%)	118 (75%)	−9.8 (−26.6 to 7.0)	0.227
Basal ganglia perivascular spaces (3–4 points)	3 (8%)	18 (11%)	−3.3 (−13.4 to 6.8)	0.562
Medial temporal lobe atrophy score (2–4)*	19 (51%)	51 (32%)	19.1 (1.3 to 36.7)	0.029
Global cortical atrophy score (2, 3)*	14 (38%)	34 (22%)	15.7 (−1.2 to 32.6)	0.048

* Indicates a statistically significant difference.

^a^ For sulcal hyperintensity in cerebral amyloid angiopathy‐related inflammation cohort *N* = 36, 1 patient had no fluid‐attenuated inversion recovery imaging available.

^b^ Contrast enhancement is carried out in 24 patients with cerebral amyloid angiopathy‐related inflammation; it is not performed for ‘non‐inflammatory’ cerebral amyloid angiopathy patients.

ARIA‐E = amyloid‐related imaging abnormalities with edema/effusion; CAA = cerebral amyloid angiopathy; CAA‐ri = cerebral amyloid angiopathy‐related inflammation; IQR = interquartile range.

#### Boston 2.0 Diagnostic Criteria for “Non‐Inflammatory” Sporadic CAA

**FIGURE 1:**
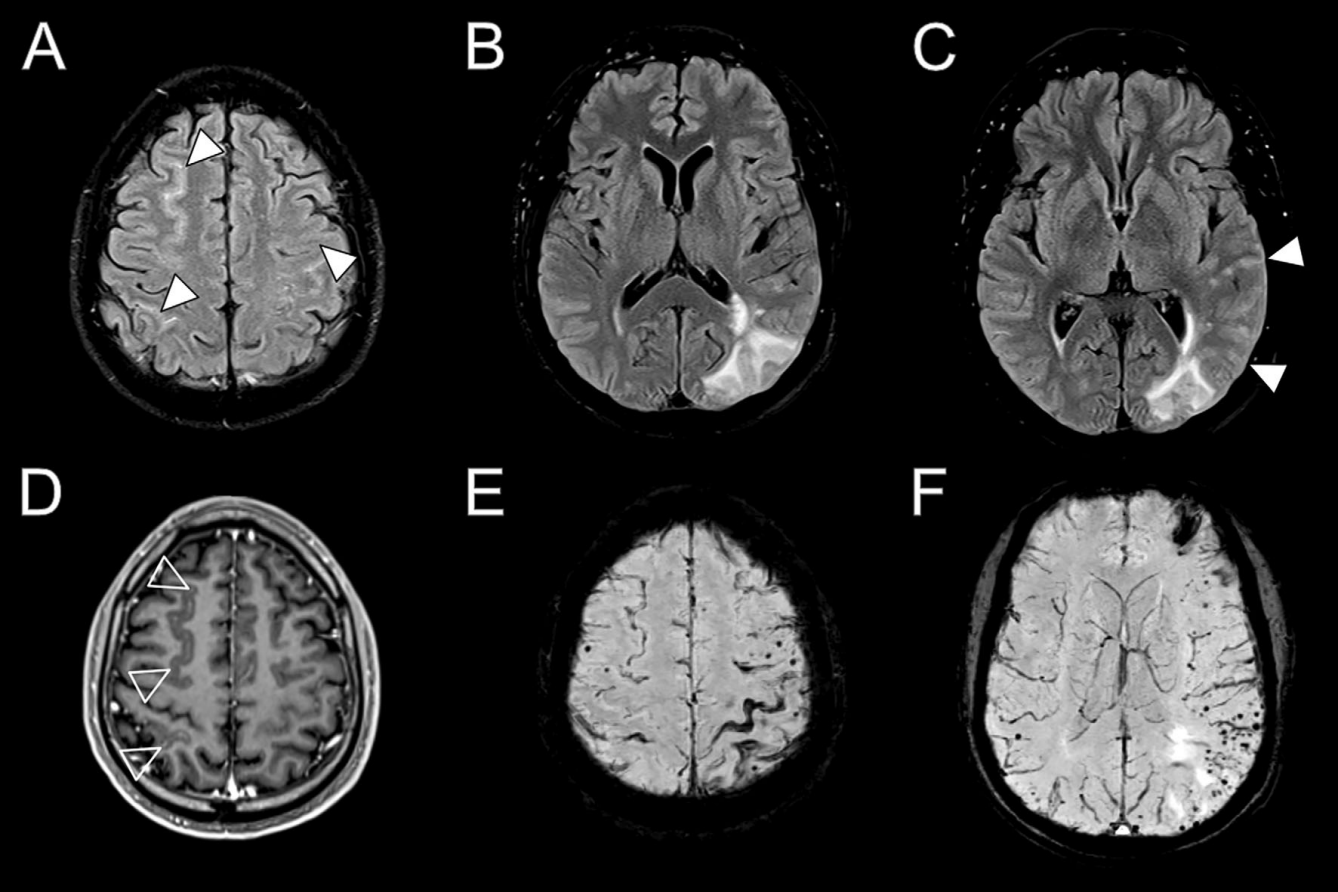
Representative magnetic resonance imaging biomarkers of cerebral amyloid angiopathy‐related inflammation. (A–C) Fluid attenuated inversion recovery hyperintensity imaging of the same patient demonstrated (A) convexity sulcal hyperintensities (white arrows), (B, C) left parieto‐occipital white matter hyperintensities, and (C) gyral swelling (white arrows). (D) Contrast‐enhanced T1‐weighted image showed leptomeningeal enhancement (arrowheads). (E, F) Susceptibility‐weighted imaging showed extensive superficial siderosis that was most apparent in the left central sulcus and over the (E) left frontal pole, and (F) numerous lobar microhemorrhages.

Lobar cerebral microbleeds were much more numerous in patients with CAA‐ri (median CMB count 207 [IQR 33–811] vs 19 [IQR 7–58], *p* < 0.001). However, other hemorrhagic markers were more frequently observed in patients with “non‐inflammatory” sporadic CAA than those with CAA‐ri, including acute and previous intracerebral hemorrhage (54% vs 22% in CAA‐ri, *p* < 0.001), convexity subarachnoid hemorrhage (28% vs 11%, *p* = 0.030), and cortical superficial siderosis (74% vs 38%, *p* < 0.001). Example images for hemorrhagic markers are provided in Figure 1E and F.

When considering non‐hemorrhagic markers, there were no significant between‐group differences in the prevalence of >20 visible enlarged perivascular spaces in the centrum semiovale (65% of patients with CAA‐ri vs 75% of patients with “non‐inflammatory” sporadic CAA, *p* = 0.113) or “multispot” white matter hyperintensities (30% of CAA‐ri patients vs 47% of “non‐inflammatory” sporadic CAA, *p* = 0.059).

#### ARIA‐E

##### Parenchymal White Matter Hyperintensities

Confluent parenchymal white matter hyperintensities were present in 33 of 37 (89%) patients with CAA‐ri at onset, compared with smaller confluent parenchymal hyperintensities in only 5 (3%) of the patients with “non‐inflammatory” sporadic CAA (*p* < 0.001). There was evidence of mass effect in 29 out of 33(88%) of these patients with CAA‐ri and parenchymal white matter hyperintensities. The hyperintensities were bilateral at presentation in 52% of cases and multifocal in 59%, with involvement of ≥3 lobar regions in 48%. Example images are provided in Figure 1B and C.

##### Sulcal Hyperintensities

We observed sulcal hyperintensities in 28 of 36 (78%) patients with CAA‐ri (1 patient did not have FLAIR MRI image at the onset). These were was bilateral in 9 of 28 (32%) patients, and involved ≥3 lobar regions in 8 of 28 (29%) patients. In most cases (18/28; 64%), sulcal hyperintensities were adjacent to areas of parenchymal white matter hyperintensities. Example images are provided in Figure 1A. By contrast, sulcal hyperintensities were observed in only 15 out of 158 (9%, *p* < 0.001) of the patients with “non‐inflammatory” sporadic CAA.

##### Gyral Swelling

Gyral swelling was observed in 32 of 37 (86%) patients with CAA‐ri, compared with 1 of 158 (0.6%, *p* < 0.001) patients with “non‐inflammatory” sporadic CAA. In 69% of patients (*n* = 22), gyral swelling colocalized with parenchymal white matter hyperintensities, particularly with hyperintensities associated with mass effect. In 3 patients, we observed colocalization of sulcal hyperintensity and gyral swelling in the absence of parenchymal white matter hyperintensities. Example images are provided in Figure 1C. Focal gyral swelling colocalized with sulcal hyperintensity was observed in only 1 patient with “non‐inflammatory” CAA.

The BGTS MRI scores for ARIA‐E are presented in Table S1.

#### Other Radiological Observations

##### Contrast Enhancement

Contrast‐enhanced MRI was performed in 24 of 37 (65%) patients with CAA‐ri, and leptomeningeal enhancement was observed in 17 of 24 (71%) of these patients. It was unilateral in 10 of 17(59%) and bilateral in 7 of 17(41%) patients. Leptomeningeal contrast enhancement was colocalized or partially overlapped with gyral swelling in 15 of 17(88%) patients, and with sulcal hyperintensities in 12 of 17 (71%) patients. Example images are provided in Figure 1D. Contrast‐enhanced MRI was not routinely performed for patients with “non‐inflammatory” sporadic CAA.

#### Neuroimaging Markers of Atrophy

Patients with CAA‐ri were more likely to have atrophy on brain imaging than patients with “non‐inflammatory” sporadic CAA. For medial temporal atrophy, measured using the Scheltens' visual scale,^24^ 19 of 37 (51%) patients with CAA‐ri had scores ≥2 on the most affected side (suggestive of clinically significant atrophy) compared with 51 of 158(32%) patients with “non‐inflammatory” sporadic CAA (*p* = 0.029). Global cortical atrophy, measured using the Pasquier visual scale,^25^ was also more frequent in patients with CAA‐ri than in “non‐inflammatory” sporadic CAA (score ≥2 in 38% vs 22%, respectively, *p* = 0.048). The exact medial temporal atrophy and global cortical atrophy score distributions are shown in Table S2.

**FIGURE 2:**
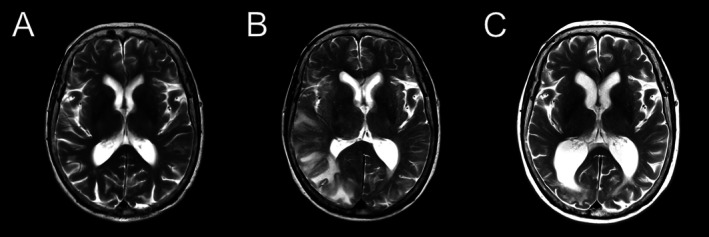
Brain volume loss after cerebral amyloid angiopathy‐related inflammation on serial T2 magnetic resonance imaging. (A) T2 images of the same patient showed a brain image 1 year before the onset of inflammation; (B) the image at the time of active inflammation; and (C) the follow‐up image 7 months later, showing volume loss with *ex vacuo* dilatation of the trigone of the right lateral ventricle.

Among those CAA‐ri patients with follow‐up imaging (*n* = 37), 6 (16%) developed marked generalized brain volume loss after their first inflammatory episode had resolved, which, on expert neuroradiological review, did not appear to be explained by the resolution of edema alone or preexisting conditions (only 1 patient had a previous disabling stroke). Example images of the evolution of marked brain atrophy are provided in Figure 2.

## Discussion

Our key findings are: (1) imaging features of ARIA‐E (ie, asymmetric parenchymal white matter hyperintensities, sulcal hyperintensities, or gyral swelling) are common in patients with CAA‐ri, but rare in patients with “non‐inflammatory” sporadic CAA; (2) patients with CAA‐ri are much more likely to have very numerous cerebral microbleeds, medial temporal and global cortical atrophy, imaging features suggestive of capillary CAA, Alzheimer's disease (ie, parenchymal Aβ and/or tau), or both, compared with patients with “non‐inflammatory” sporadic CAA; and (3) patients with CAA‐ri were more likely than those with “non‐inflammatory” sporadic CAA to have conventional vascular risk factors and other long‐term non‐vascular medical conditions (ie, long‐term inflammatory or infectious conditions, autoimmune or connective tissue conditions, or malignancy). These findings have potentially important diagnostic and mechanistic implications, with additional relevance for evaluating and managing CAA‐ri within a clinical service.

Our findings suggest that there are shared core imaging findings in two different Aβ‐related neuroinflammatory conditions (ARIA‐E and CAA‐ri), further supporting the hypothesis that CAA‐ri might be a spontaneously occurring model of the ARIA‐E associated with Alzheimer's disease immunotherapy.^26^ Indeed, consistent with this hypothesis, we recently reported sulcal hyperintensities ‐ a feature of ARIA‐E ‐ as the earliest imaging finding in CAA‐ri, preceding classical white matter hyperintensities.^16^ The particularly high prevalence of sulcal hyperintensities and gyral swelling in CAA‐ri, in addition to the already well‐recognized parenchymal white matter hyperintensities, suggests that all of these radiological features should be routinely assessed in suspected CAA‐ri. Inclusion of these neuroimaging features (ie, sulcal hyperintensities and gyral swelling) in future iterations of diagnostic criteria for CAA‐ri could improve their sensitivity and specificity, particularly in those patients with atypical, mild or early presentations, who may still currently undergo brain biopsy for diagnosis. Although these core imaging features (sulcal hyperintensities in particular) were occasionally observed in patients with “non‐inflammatory” sporadic CAA, their evaluation in other inflammatory disorders which can resemble CAA‐ri (for example, cerebral vasculitides or encephilitides) will be essential in guiding future changes to diagnostic criteria.^27^ Because increased signal in brain sulci on FLAIR images can be caused not only by sulcal hyperintensities, but also by acute convexity subarachnoid hemorrhage, careful comparison of FLAIR and blood‐sensitive images is important to avoid false overestimation of the latter. Full application of the ARIA‐E BGTS involves rating across multiple brain regions, with a resulting score between 0 and 60; the correlation of this score with clinical severity and prognosis (including risk of relapse) merits further study in CAA‐ri.

We also found strikingly different profiles of neuroimaging markers of hemorrhage and atrophy in patients with CAA‐ri compared with those with “non‐inflammatory” sporadic CAA. There was a much greater number of lobar cerebral microbleeds (but less intracerebral hemorrhage, convexity subarachnoid hemorrhage, and cortical superficial siderosis), and higher rates of both medial temporal lobe and generalized cortical atrophy in CAA‐ri compared with “non‐inflammatory” sporadic CAA.

The greater amount of ICH, convexity subarachnoid hemorrhage, and cortical superficial siderosis in the sporadic “non‐inflammatory” group may reflect the specifics of patient selection, as patients with hemorrhagic manifestations of CAA are mainly referred to the clinic. However, the high number of lobar CMB in the CAA‐ri group could represent two possible pathophysiological mechanisms: first, inflammation potentiates blood–brain barrier disruption, which could trigger CMB formation, leading to a high number of CMBs in CAA‐ri;^28^ or alternatively, a difference in the type of predominant CAA pathology. Histopathological and radiological data^29^, ^30^ suggest that there are 2 distinct forms of CAA: type 1, in which there is cortical capillary Aβ deposition (as well as other cortical and leptomeningeal arterial and venous vessels), and type 2, in which cortical capillary involvement is absent, with Aβ deposition primarily affecting leptomeningeal and cortical arteries (and to a lesser extent, leptomeningeal and cortical veins).^31^ Type 1 CAA appears to be associated with numerous lobar CMB, the presence and severity of medial temporal lobe atrophy, and the apolipoprotein E (ApoE) ε4 allele (a recognized risk factor for Alzheimer's disease).^32^, ^33^, ^34^ Our findings therefore could suggest that type 1 (capillary CAA), with or without neurodegeneration associated with parenchymal Alzheimer's disease pathology, might be a risk factor for CAA‐ri. The ApoE ε4 allele is a recognized risk factor for developing ARIA,^35^, ^36^ with data from a small case series suggesting a similar increased risk for CAA‐ri.^4^, ^37^ It is tempting to speculate that capillary CAA or parenchymal Aβ deposition, or both, might be important in driving the pathophysiology of CAA‐ri, although this will need further investigation with histopathological studies and through ApoE genotyping in larger CAA‐ri cohorts. If the relevance of parenchymal amyloid, capillary CAA, or both can be confirmed in other studies, this would support the emerging concept that CAA‐ri is indeed a spontaneous model of the ARIA‐E observed in Aβ immunotherapy trials in AD.

However, the medial temporal atrophy, and particularly the global cortical atrophy score, as imaging markers in Alzheimer's disease, are not entirely specific. Brain volume reduction is observed in various types of small vessel disease, and the level of brain atrophy is often utilized as a surrogate marker for disease progression.^38^ Nevertheless, they remain useful markers for neurodegeneration, especially the medial temporal atrophy score.^39^ Integrating these markers with clinical, cognitive, and other biomarker data in CAA‐ri or CAA might enhances the accuracy of diagnosis and understanding of underlying pathophysiological mechanisms.

The time course of atrophy developing in CAA‐ri might also be relevant (ie, whether before or after the CAA‐ri inflammatory episode). In addition to atrophy at presentation, we also observed new atrophy in 16% of patients with CAA‐ri who had follow‐up brain MRI available. Atrophy in patients with Alzheimer's disease successfully treated with Aβ immunotherapies is recognized, with some proposing this “amyloid removal‐related pseudo‐atrophy” is a physiological (rather than pathological) response to plaque removal^40^; the contributions of ARIA‐E in driving this “pseudo‐atrophy” remain unclear, but our findings raise the possibility that a similar phenomenon of volume loss related to amyloid elimination from the brain might occur in CAA‐ri.

Although outcomes in CAA‐ri have largely focused on the risk of inflammatory relapse, further investigation into whether patients with CAA‐ri are more likely to develop cognitive decline—rather than recurrent intracranial hemorrhage—could help define their long‐term clinical trajectory in comparison with those with sporadic CAA.

We observed that, compared with patients with “non‐inflammatory” sporadic CAA, patients with CAA‐ri were more likely to have both conventional vascular risk factors (hypercholesterolemia, hypertension) and non‐vascular long‐term medical conditions, including those associated with persistent systemic inflammatory states, autoimmunity or connective tissue diseases, or solid organ or hematological malignancies. Although we are not aware of any systematic investigation of this association in cohort studies of patients with CAA‐ri, on review of the existing literature, non‐vascular long‐term medical conditions are indeed a recognized observation in CAA‐ri, with published reports describing CAA‐ri in patients with autoimmune thyroiditis,^41^ rheumatoid arthritis,^13^, ^41^, ^42^ sarcoidosis,^43^ psoriasis,^44^ giant cell arteritis,^45^ and granulomatous polyangiitis,^46^ among others. However, these case reports could be subject to ascertainment bias (ie, unusual cases are more likely to be reported). Our findings in a larger and more representative cohort study suggest that these vascular risk factors and non‐vascular long‐term conditions might be risk factors for CAA‐ri. The underying mechanisms could include disruption of vascular integrity, immune regulation, or both, thereby potentially increasing the likelihood of an immunogenic response to Aβ deposits.^47^ For autoimmune conditions, either the predisposition to autoimmunity or its treatment could have implications for the pathophysiology of CAA‐ri; this group also potentially requires careful diagnostic evaluation to exclude secondary brain involvement of a systemic inflammatory disorder and considered management decisions, particularly those regarding long‐term immunosuppression for both the autoimmune disease and CAA‐ri, which we recommend should be considered in a multidisciplinary setting. Recognizing and managing vascular risk factors and comorbidities in patients with CAA‐ri could potentially improve disease outcome and reduce relapse. Further research in larger cohorts is needed to understand the impact of these factors on the development and natural course of CAA‐ri.

We acknowledge limitations of this work. These are retrospective data from a single specialist center, which might not necessarily completely represent the full clinical and radiological spectrum of CAA‐ri, thus limiting the generalizability of our findings and conclusions. These data were acquired in the provision of routine clinical care, and therefore, some imaging data and data for ancillary investigations performed at the discretion of the treating physician, such as CSF studies, ApoE genotyping, blood and CSF neurodegenerative biomarkers, etc, were not available for all patients. Radiological images were reviewed without blinding to biopsy results and clinical characteristics, which is also a potential source of bias. We acknowledge that the patients with CAA‐ri that were referred to our specialist service might have more severe disease (and possibly more cerebral microbleeds) than those with CAA‐ri who were not referred, potentially introducing selection bias; additonally, patients with vasogenic edema, but less typical imaging features of CAA, such as minimal hemorrhagic biomarkers (CMB, no cortical superficial siderosis), may have been misdiagnosed with vasculitis or other mimics of CAA‐ri.

Despite these limitations, our report provides a detailed characterization of a CAA‐ri patient cohort, with clinical and radiological findings that could be of mechanistic, diagnostic, and prognostic relevance. Our findings suggest a widening clinical and neuroimaging CAA‐ri phenotype, which emphasizes similarities with the ARIA‐E observed in Alzheimer's disease immunotherapy trials. Capillary CAA, parenchymal Aβ, or both, might be key risk factors for CAA‐ri, in addition to long‐term inflammatory, autoimmune conditions, or malignancies, which could increase patient susceptibility to CAA‐ri. However, confirmation of these observations by other centers will be important, indicating an unmet need for national and international efforts to centralize CAA‐ri reporting across specialist and non‐specialist centers; this will be critical for acquiring less biased natural history data and could also provide a platform for large multicenter treatment trials.

## Author Contributions

L.P. and D.J.W. contributed to the conception and design of the study; L.P., G.B., D.M., K.T., R.O., V.H., G.A., M.Z., and H.R.J. contributed to the acquisition and analysis of data; L.P., G.B., D.M., and D.J.W. contributed to drafting the text or preparing the figures.

## Potential Conflicts of Interest

Nothing to report.

## Supporting information


**Supplemental Table S1.** The median (IQR) Barkhof Grand Total Scale (BGTS) MRI score for ARIA‐E in patients with CAA‐ri.
**Supplemental Table S2.** The exact enlarged perivascular spaces in the centrum semiovale (EPVS‐CSO) score, medial temporal atrophy (MTA) and global cortical atrophy (GCA) score distributions.

## Data Availability

The author has full access to the data used in this manuscript. Patient data collected as part of a service evaluation are not the subject of disclosure.
