# Supplementary material for: Clinical–Radiological Spectrum of Cerebral Amyloid Angiopathy‐Related Inflammation
Source: Ann Neurol. 2025 Sep 19;99(1):148–58. doi: 10.1002/ana.78029 (PMC12946602; doi:10.1002/ana.78029)
Supplement: Supplementary file 1 — Supplemental Table S1. The median (IQR) Barkhof Grand Total Scale (BGTS) MRI score for ARIA‐E in patients with CAA‐ri. Supplemental Table S2. The exact enlarged perivascular spaces in the centrum semiovale (EPVS‐CSO) score, medial temporal atrophy (MTA) and global cortical atrophy (GCA) score distributions. [file ANA-99-148-s001.docx]

Supplemental Table 1. **The median (IQR) Barkhof Grand Total Scale (BGTS)** **MRI** **score for ARIA-E in patients with CAA-ri.**

|  | Frontal lobe | | Temporal lobe | | Parietal lobe | | Occipital lobe | |
| --- | --- | --- | --- | --- | --- | --- | --- | --- |
|  | R | L | R | L | R | L | R | L |
| PH | 0 (0-1.25) | 0 (0-1) | 0 (0-4) | 1 (0-3) | 1 (0-4) | 0 (0-2.25) | 1 (0-3) | 0 (0-3) |
| SH | 0 (0-0) | 0 (0-0) | 0 (0-1) | 0 (0-0.5) | 0 (0-0.5) | 0 (0-0) | 0 (0-1.5) | 0 (0-1) |
| GS | 0 (0-0.5) | 0 (0-0) | 0 (0-4) | 0 (0-3) | 0.5 (0-4) | 0 (0-2.25) | 0 (0-3) | 0 (0-1.25) |
| Regional score | 0 (0-3) | 0 (0-1.25) | 1 (0-4) | 2 (0-4) | 3 (0-4) | 0 (0-4) | 3 (0-4.25) | 2 (0-3) |
| Total score | 10.5 (7-24.5) | | | | | | | |

Abbreviations: PH – parenchymal white matter hyperintensities, SH – sulcal hyperintensities, GS – gyral swelling.

Supplemental Table 2. **The exact enlarged perivascular spaces in the centrum semiovale (EPVS-CSO) score,** **medial temporal atrophy** (**MTA) and global cortical atrophy (GCA) score distributions.**

|  | CAA-ri (n=37) | CAA (n=158) |
| --- | --- | --- |
| EPVS-CSO score, n (%)  0  1  2  3  4 | 1 (2%)  4 (11%)  8 (22%)  7 (19%)  17 (46%) | 1 (0.6%)  15 (9%)  25 (16%)  38 (24%)  80 (51%) |
| MTA score, n (%)  0  1  2  3  4 | 5 (14%)  13 (35%)  5 (14%)  12 (32%)  2 (5%) | 37 (23%)  70 (44%)  32 (21%)  16 (10%)  3 (2%) |
| GCA score, n (%)  0  1  2  3 | 7 (19%)  16 (43%)  10 (27%)  4 (11%) | 38 (24%)  85 (54%)  28 (18%)  7 (4%) |
